# RETRACTION: Mendelian randomization supports a causative effect of TSH on thyroid carcinoma

**DOI:** 10.1530/ERC-20-0067r

**Published:** 2020-11-14

**Authors:** Jonathan M Fussey, Robin N Beaumont, Andrew R Wood, Bijay Vaidya, Joel Smith, Jessica Tyrrell

**Affiliations:** 1 Head and Neck Surgery, Royal Devon and Exeter Hospital, Exeter, UK; 2 Genetics of Complex Traits, Institute of Biomedical and Clinical Science, University of Exeter Medical School, Exeter, UK; 3 Endocrinology, Royal Devon and Exeter Hospital, Exeter, UK

This article has been retracted by the journal.

The article has been retracted because *Endocrine-Related Cancer* was made aware, by the corresponding author J M Fussey, of an issue with the data analysis, which affected the results and conclusions of the manuscript ‘Mendelian randomization supports a causative effect of TSH on thyroid carcinoma’ published on 18 August 2020 in volume 27 part 10, pages 551–559. As such, and with agreement from all authors, the article has now been retracted. No further action will be taken against the authors of the article.

This retraction was published 23 October 2020.

